# Characterization of the complete chloroplast genome sequence of *Diospyros kaki* cv. *Luotiantianshi*

**DOI:** 10.1080/23802359.2021.1972869

**Published:** 2021-09-15

**Authors:** Yang Xu, Kai-Yun Wu, Bang-chu Gong

**Affiliations:** Research Institute of Subtropical Forestry, Chinese Academy of Forestry, Hangzhou, China

**Keywords:** *Diospyros* kaki cv. *Luotiantianshi*, chloroplast genome, phylogenetic analysis

## Abstract

*Diospyros kaki* cv*. Luotiantianshi* is a rare germplasm of *Diospyros* Linn in the world. In this study, we generated the complete chloroplast (cp) genome of *D*. *kaki* cv. *Luotiantianshi.* The complete cp genome was 157,773 bp in length, containing a large single copy region (LSC) of 87,066 bp, a small single copy region (SSC) of 18,529 bp, and two inverted repeat (IR) regions of 26,089 bp. The new sequence has a total of 131 genes, including 86 protein-coding genes, 37 tRNA genes, and 8 rRNA genes. Further, phylogenetic analysis showed that the *D*. *kaki* cv. *Luotiantianshi* has a close relationship with *Diospyros kaki*. This study provides important information for future evolution, genetic and molecular biology studies of *Diospyros*.

*Diospyros kaki* Thunb 1780 cv. *Luotiantianshi*, also is called Chinese PCNA (pollination-constant non-astringent) type persimmon (*Diospyros kaki* Thunb 1780), only distributes in Luotian and Macheng of Hubei province. *Diospyros kaki* cv. *Luotiantianshi*, which is morphologically and genetically different from Japanese pollination constant and non-astringent (JPCNA) or other persimmons by natural de-astringency model of fruits (Yonemori et al. 2005), is a rare germplasm of *Diospyros* Linn in the world (Zhang et al. 2016). In the present study, the complete chloroplast genome of *D. kaki* cv. *Luotiantianshi* was obtained using the next-generation sequencing (NGS) technologies and a phylogenetic analysis of this species and its relatives was carried out.

Total DNA was extracted from 5 g fresh leaves gathered from Pinghu town in Luotian county, Huanggang city, Hubei province of China (115°22'2″E, 30°54'49″N) using CTAB method (Doyle and Doyle 1987). A specimen was deposited at Research Institute of Subtropical Forestry, Chinese Academy of Forestry (URL: http://risfcaf.caf.ac.cn/, contact person: Bang-chu Gong, email: gongbc@126.com) under the voucher number: MBLS-LTTS84. The DNA libraries was constructed and sequenced on Illumina HiseqXten platform (Illumina, San Diego, CA) for 150 bp paired-end sequencing by Biodata Biotechnologies Inc. (Hefei, China). The cp genome was assembled using a *de novo* strategy according to the work of Xu et al. (2020).

The size of the complete chloroplast genome of *D. kaki* cv. *Luotiantianshi* was 157,773 bp, which was smaller than *D. kaki* (157,784 bp) and larger than *Diospyros oleifera* Cheng 1935 (157,724 bp) (Fu et al. 2016). The cp genome of *D. kaki* cv. *Luotiantianshi* was composed of a large single-copy region (LSC, 87,066 bp), a small single-copy region (SSC, 18,529 bp), and two inverted repeats (IR, 26,089 bp). The total GC content of new chloroplast genome was 37.4%, and while it was 43.1% of IRs, which was higher than LSC and SSC regions (35.40% and 30.80%, respectively). The new sequence has 86 protein-coding genes, was one less than that in *D. kaki* (87), 37 tRNA genes, and 8 rRNA genes.

A phylogenetic analysis was carried out using whole cp genome sequences of *D. kaki* cv. *Luotiantianshi* and other 14 species in *Diospyros*. Meanwhile, two species from the *Actinidia* (*Actinidia arguta* Sieb. & Zucc 1864 and *Actinidia chinensis* Planch 1847) were used as outgroups. The sequences were aligned using MAFFT version 7 (Kazutaka and Standley 2013). The maximum-likelihood (ML) method base on GTR + G + I model was used to construct the phylogenetic tree with Mega7 using 1000 bootstrap (Kumar et al. 2016). The results (Figure 1) indicated that *D. kaki* cv. *Luotiantianshi* has a close relationship with *D. kaki* under 100% bootstrap support values. The complete cp genome sequence of *D. kaki* cv. *Luotiantianshi* can be great benefit to further study on its population genetics and molecular breeding.

**Figure 1.**
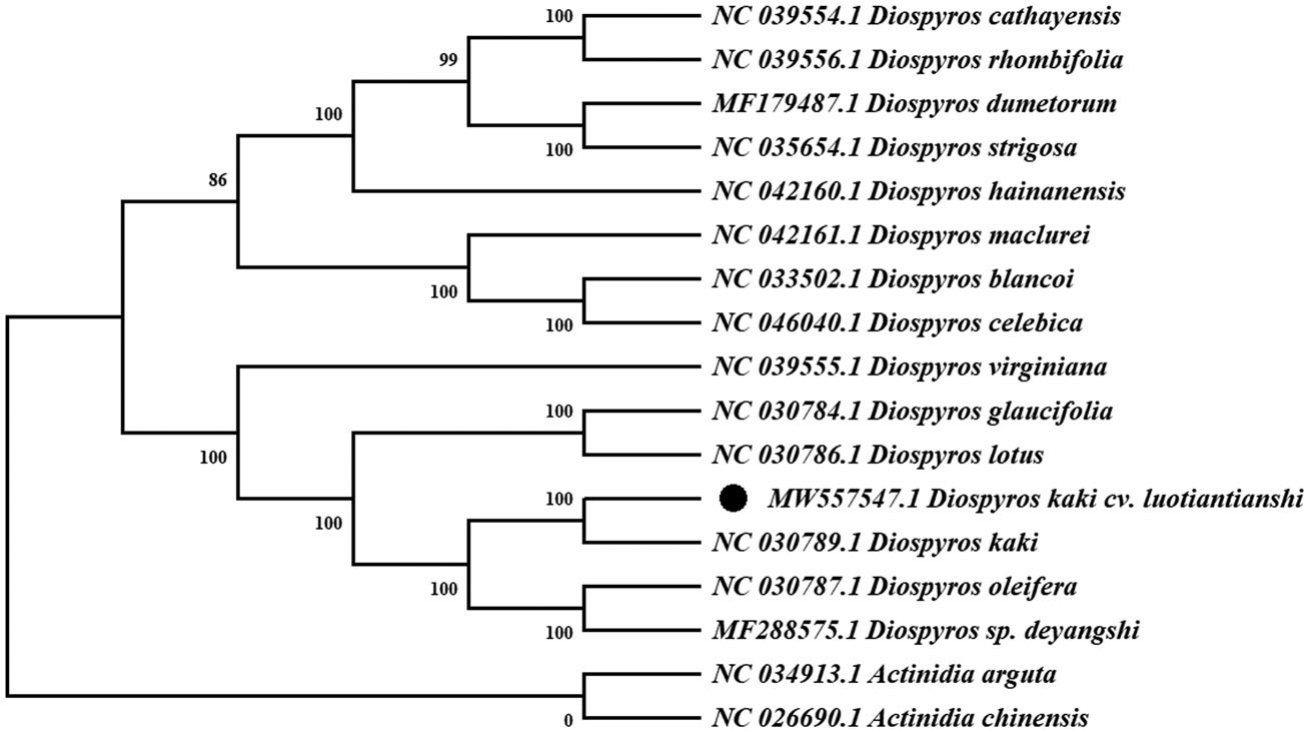
A phylogenetic tree inferred from 17 cp genomes using the maximum likelihood (ML) analysis. The position of *D. kaki* cv. *Luotiantianshi* is shown in a black solid circle and bootstrapping values are listed in each node.

## Data Availability

The genome sequence data that support the findings of this study are openly available in GenBank of NCBI at https://www.ncbi.nlm.nih.gov under the accession no. MW557547.1 The raw sequence data used in this research were deposited successfully with registered numbers of associated BioProject, SRA, and Bio-Sample:PRJNA701835, SRX10097592, and SAMN17914800, respectively
